# Dynamic-boundary-based lateral motion synergistic control of distributed drive autonomous vehicle

**DOI:** 10.1038/s41598-021-01947-3

**Published:** 2021-11-22

**Authors:** Kai Wang, Weiping Ding, Mingliang Yang, Qiao Zhu

**Affiliations:** School of Mechanical Engineering, Southwest Jiaotong University, Chengdu, 610031 Sichuan China

**Keywords:** Mechanical engineering, Electrical and electronic engineering

## Abstract

To improve the path tracking accuracy and yaw stability of distributed drive autonomous vehicles (DDAVs) under extreme working conditions, a cooperative lateral motion control method based on the dynamic boundary is proposed to prevent different road adhesion conditions from affecting the motion stability of DDAVs. Based on the analysis of the DDAV lateral dynamics system coordination mechanism, a dynamic boundary considering the pavement adhesion coefficient is proposed, and the Lateral Motion Synergistic Control System (LMSCS) is designed. The LMSCS is divided into the coordination, control, and executive layers. The coordination layer divides the control domain into the stable, quasi-stable, and unstable domains by the dynamic boundary, and coordinates the control strength of the path following control and yaw stability control. In the control layer, the path following control and yaw stability control laws are designed based on the global fast terminal sliding mode. The executive layer estimates the expected steering wheel angle and expected additional wheel torque. Joint simulations under double line shifting conditions confirmed that LMSCS reflects the impact of the road attachment conditions and improves the path tracking accuracy and vehicle yaw stability. The LMSCS has better overall performance than existing lateral motion control methods.

## Introduction

With the rapid growth of car ownership, social problems such as traffic safety, traffic congestion, and environmental pollution have emerged. Moreover, the requirements with regard to the safety, efficiency, and mobility of automobiles are constantly expanding^1, 2^, and distributed drive autonomous vehicles (DDAVs) have the characteristics of electrification, intelligence, and multi-degree-of-freedom control, which are advantageous in solving various traffic problems and satisfying users’ needs. Additionally, DDAVs combine torque vector control and path tracking control to improve the vehicle yaw stability and lateral movement safety while improving the path tracking accuracy. As the research objects of distributed drive vehicles and autonomous vehicles, torque vector control^3, 4^ and path tracking control^5, 6^ have become research hotspots in the field of DDAV lateral dynamics control^7, 8^. How the torque vector control and path tracking control work together to improve the comprehensive lateral dynamics performance of DDAV has become a challenge for researchers.

As a key technology for automatic driving, path tracking control focuses on controlling the car steering system to drive the car along the expected path while ensuring the car’s stability and travel comfort. The pure pursuit^9^ and Stanley^10^ methods are classic path tracking control algorithms based on automotive kinematics, and have simple and real-time characteristics, but do not consider the vehicle’s dynamic performance, which is generally achieved at low speed and under other simple working conditions. To adapt to the automatic vehicle’s complicated and variable driving conditions and consider the nonlinear dynamic characteristics of automatic driving vehicles, the path-based path tracking control method has been extensively investigated^11, 12^. The dynamic path tracking control method often uses a conventional linear two-degrees-of-freedom (2-DOF) model as a reference model^13, 14^, and the employed control algorithm includes the PID algorithm^15^, model prediction algorithm^16^, sliding mode control^5^, and reinforcement learning algorithm^17^.

Distributed drive vehicles achieve yaw stability control without affecting the longitudinal speed through torque vector control^18^. Many studies on the yaw stability control of distributed drive vehicles have been conducted^19–26^. The yaw speed and sideslip angle of the traditional linear 2-DOF vehicle dynamics model under steady-state steering are typically considered as the control reference targets, and the objective of improving the yaw stability of the vehicle is achieved by reducing the yaw speed and sideslip angle under complex conditions^19–26^. The typical algorithms used in Yaw Stability Control (YSC) research include PID control^19, 20^, fuzzy control^21, 22^, adaptive control^23^, sliding mode control^24, 25^, and optimization control^26^.

Although the above-mentioned path tracking control method or yaw stability control method improves the path tracking accuracy or yaw stability of DDAVs, the integrated control of Path Following Control (PFC) and YSC more effectively improves the lateral motion performance of DDAVs^7, 8, 13, 27, 28^. In^27^, the adaptive high-order control law and pseudo-inverse low-order control law are designed for DDAV path tracking control. An adaptive fuzzy sliding mode control law is designed for DDAV yaw stability control. However, less consideration is given to the coordinated control between path tracking and yaw stability. In^13^, a hierarchical control scheme is proposed to improve the trajectory tracking ability and lateral stability of DDAVs, and the working areas of PFC and YSC are coordinated based on the working conditions of tires. In^28^, a robust DDAV path following control strategy based on the nonsingular terminal sliding mode and active disturbance rejection control is presented. The simulation results obtained under the double line change condition reveal that the proposed control strategy ensures that the vehicle quickly and accurately follows the reference path, and has strong robustness. Currently, there are many algorithms for PFC and YSC integrated control. The sliding mode control has the advantages of fast response, being insensitive to parameter changes, no online identification of the system, and simple physical implementation^29^, which make sliding mode control suitable to vehicle dynamic performance control. Therefore, sliding mode control has been extensively investigated, and many new control algorithms based on sliding mode control theory, such as Global Fast Terminal Sliding Mode (GFTSM)^30, 31^, have emerged. The GFTSM algorithm has the following features: the system can converge to the equilibrium state within a limited amount of time, and the convergence time can be designed. The continuous control law is adopted, and does not include a switching term, which is beneficial for reducing the chattering phenomenon of sliding mode control. The GFTSM has good robustness to system uncertainties and disturbances^29^, and should be further applied to practical DDAV dynamic performance control.

This paper proposes a DDAV coordinated control method based on the dynamic boundary, and a DDAV coordinated control system is designed. The main contributions of this study are as follows: (1) In existing studies, the yaw velocity and sideslip angle of the traditional linear 2-DOF vehicle dynamics model under steady-state steering are typically considered as reference targets for stability control, without considering the road conditions. To solve this problem, a 2-DOF vehicle dynamics model with a road adhesion coefficient is designed. Based on the dynamic model and tire force limit, the dynamic boundary describing the stable state of DDAV lateral movement is derived to consider the influence of additional variables. The control domain is divided into a stable, quasi-stable, and non-stable region through the dynamic boundary to more accurately identify the stable state of vehicle lateral movement. (2) Based on the recognition of DDAV’s stable state of lateral motion, a coordinated control law for PFC and YSC is constructed to fully active the DDAV's multi-degree-of-freedom control advantage such that LMSCS could improve the path tracking performance and vehicle yaw stability simultaneously. (3) The GFTSM-based wheel angle control law and additional yaw moment control law are designed. Compared with the existing lateral motion control method based on the traditional sliding mode algorithm, it is demonstrated that the adoption of GFTSM in LMSCS can achieve better lateral motion control. (4) The effectiveness and superiority of the proposed DDAV lateral motion co-control method based on the dynamic boundary are verified by simulation under different vehicle speeds, road adhesion coefficients, and double lane shifting conditions.

The rest of this paper is organized as follows: In “Dynamic system analysis” section, the vehicle dynamics model based on the design and validation of LMSCS is analyzed. In “Control system design” section, the sub-strategy design method of DDAV lateral motion cooperative control system based on the dynamic boundary is described. In “Demonstrative example” section, the proposed DDAV lateral motion cooperative control method based on the dynamic boundary is applied to an example DDAV model, and the effectiveness and superiority of the designed LMSCS are demonstrated through simulation analysis under various working conditions. “Conclusion” section, summarizes the conclusions drawn from this study.

## Dynamic system analysis

The dynamic-boundary-based LMSCS is a model-based control strategy. The premise of LMSCS design is to construct a suitable DDAV dynamic model and analyze the dynamic cooperative control mechanism of PFC and YSC to provide the theoretical basis for realizing synergistic control.

### Construction of path following vehicle dynamics model

In path tracking control, the vehicle dynamics model should be able to describe the DDAV lateral motion, yaw motion, and relationship between the heading angle and above-mentioned motions. Therefore, the 2-DOF linear vehicle dynamics model is adopted. This model has a simple expression and reflects the most basic characteristics of the car’s curve motion, and can be used to calculate the main parameters reflecting the vehicle’s lateral motion performance. The model satisfies the design requirements of the path tracking control law and ensures that the real-time performance of the algorithm is satisfactory.

The differential equations of the 2-DOF vehicle motion are as follows^32^:

1 $$\fancyscript{m}\left({\dot{V}}_{\mathcal{Y}}+{V}_{\mathcal{X}}\gamma \right)={C}_{\mathcal{Y}f}\left(\beta +\frac{a}{V}\gamma -\delta \right)+{C}_{\mathcal{Y}r}\left(\beta -\frac{b}{V} \gamma \right)$$

2 $${I}_{z}\dot{\gamma }=a{C}_{\mathcal{Y}f}\left(\beta +\frac{a}{V}\gamma -\delta \right)-b{C}_{\mathcal{Y}r}\left(\beta -\frac{b}{V} \gamma \right)$$

where $\fancyscript{m}$ is the mass of the entire vehicle; ${V}_{\mathcal{X}}$ is the longitudinal vehicle speed; ${V}_{\mathcal{Y}}$ is the lateral vehicle speed; $\gamma$ is the yaw rate; ${C}_{\mathcal{Y}f}$ is the sum of the front axle tires corner stiffness; ${C}_{\mathcal{Y}r}$ is the sum of the rear axle tires corner stiffness; $\delta$ is the average turning angle of the front wheels; $\beta$ is the sideslip angle of the DDAV; a and b are the distances from the front and rear axles to the center of mass, respectively; $V$ is the vehicle speed.

**Figure 1:**
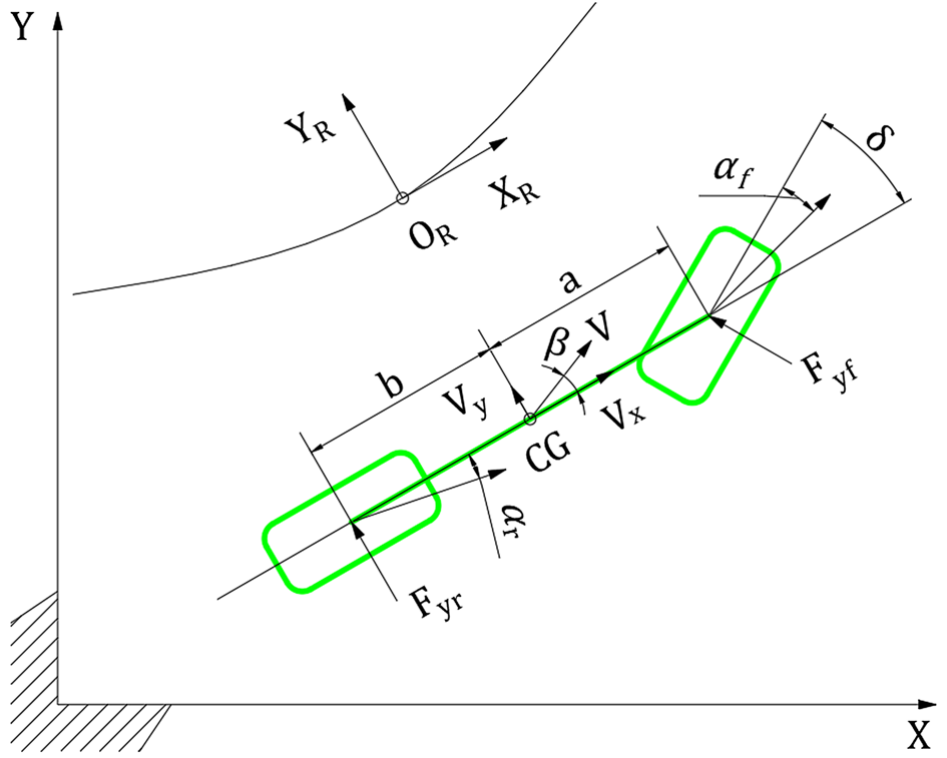
DDAV path tracking dynamic model.

Path tracking control mainly aims to limit the lateral deviation between the vehicle and given reference path. To facilitate the description of the relationship between the vehicle and the road, the road coordinate system is established as follows: The origin ${O}_{R}$ of the road coordinate system is the current path reference point, the ${X}_{R}$ axis points to the driving direction along the path tangent of the current path reference point, and the ${Z}_{R}$ axis is perpendicular to the ground in the upward direction, which conforms to the principle of the right-handed coordinate system. The current path reference point refers to the point currently closest to the center of mass of the vehicle on a given reference path. The path tracking dynamics model should also reflect the relationship between the vehicle and the road, the simplification of the DDAV to 2-DOF, and driving in the geodetic coordinate system, as shown in Fig. 1, where ${\alpha }_{f}$ is the average front wheel slip angle and ${\alpha }_{r}$ is the average rear wheel slip angle.

The DDAV speed along the ${Y}_{R}$ axis is expressed as follows:

3 $$\dot{{Y}_{R}}={V}_{\mathcal{X}}{\sin}{\psi }_{R}+{V}_{\mathcal{Y}}{\cos}{\psi }_{R}$$

where ${Y}_{R}$ is the lateral displacement of the vehicle in the road coordinate system, and ${\psi }_{R}$ is the yaw angle of the vehicle’s longitudinal direction relative to the ${X}_{R}$-axis of the road coordinate system.

### Construction of yaw stability control vehicle dynamics model

To construct the DDAV yaw stability control law, a 2-DOF vehicle dynamics model, which contains the tire force of each wheel and an additional yaw torque that reflects the lateral and yaw motion of the vehicle, respectively, is established as follows^32^:

4 $$\fancyscript{m}\left({\dot{V}}_{\mathcal{Y}}+{V}_{\mathcal{X}}\gamma \right)=\left({F}_{\mathcal{Y}fr}+{F}_{\mathcal{Y}fl}\right){\cos}\delta +\left({F}_{\mathcal{X}fr}+{ F}_{\mathcal{X}fl}\right){\sin}\delta +{F}_{\mathcal{Y}rl}+{F}_{\mathcal{Y}rr}$$

5 $$\begin{aligned} {I}_{z}\dot{\gamma } & =a\left({F}_{\mathcal{Y}fr}+{F}_{\mathcal{Y}fl}\right){\cos}\delta +\frac{{d}_{f}}{2}\left({F}_{\mathcal{Y}fl}-{F}_{\mathcal{Y}fr}\right){\sin}\delta - b\left({F}_{\mathcal{Y}rl}+{F}_{\mathcal{Y}rr} \right)+\frac{{d}_{f}}{2}\left({F}_{\mathcal{X}fr}-{F}_{\mathcal{X}fl}\right){\cos}\delta \\ & \quad + a\left({F}_{\mathcal{X}fr}+{F}_{\mathcal{X}fl}\right){\sin}\delta +{\frac{{d}_{r}}{2}\left({F}_{\mathcal{X}rr}-{F}_{\mathcal{X}rl}\right)+\Delta M}_{z} \end{aligned}$$

where ${F}_{\mathcal{X}ij}$ is the longitudinal force of the tire, ${F}_{\mathcal{Y}ij}$ is tire lateral force, ${d}_{f}$ is the front wheel base, ${d}_{r}$ is the rear wheel base, and ${\Delta M}_{z}$ is the additional yaw moment calculated by the control layer of YSC.

The tire force contained in the 3-DOF vehicle dynamics model can be calculated using the Dugoff nonlinear tire model^33^, which is an analytical model derived from the force balance relationship, has fewer custom parameters, expresses the nonlinear characteristics of the tire, and is widely used for vehicle lateral motion control^34, 35^.

The longitudinal tire force is expressed as follows:

6 $${F}_{\mathcal{X}ij}={C}_{\mathcal{X}ij}\frac{{\uplambda }_{ij}}{1+{\uplambda }_{ij}}f\left({\upsigma }_{ij}\right)$$

The lateral tire force is expressed as follows:

7 $${F}_{\mathcal{Y}ij}={C}_{\mathcal{Y}ij}\frac{{\tan}{\alpha }_{ij}}{1+{\uplambda }_{ij}}f\left({\upsigma }_{ij}\right)$$

where ${C}_{\mathcal{X}ij}$ is the tire’s longitudinal slip stiffness, ${C}_{\mathcal{Y}ij}$ is tire sideslip stiffness, ${\uplambda }_{ij}$ is the wheel slip rate, and ${\alpha }_{ij}$ is the sideslip angle of the tire (in the expression form $\mathcal{X}ij$, pin $i$ represents the front wheel or rear wheel, and $j$ represents the left wheel or right wheel).

The expression of $f\left({\upsigma }_{ij}\right)$ is as follows:

8 $${\text{f}}\left({\upsigma }_{ij}\right)=\left\{\begin{array}{ll}\left(2-{\upsigma }_{ij}\right){\upsigma }_{ij}, & \quad {\upsigma }_{ij}<1\\ 1, & \quad {\upsigma }_{ij}\ge 1\end{array}\right.$$

9 $${\upsigma }_{ij}={C}_{\mathcal{Y}ij}\frac{\mu {F}_{zij}\left(1+{\uplambda }_{ij}\right)\left[1-{ A}_{s}R{\omega }_{ij}\sqrt{{{\uplambda }_{ij}}^{2}+{{\tan}}^{2}{\alpha }_{ij}}\right]}{2\sqrt{{\left({C}_{\mathcal{X}ij}{\uplambda }_{ij}\right)}^{2}+{\left({C}_{\mathcal{Y}ij}{\tan}{\alpha }_{ij}\right)}^{2}}}$$

where $\mu$ is the pavement adhesion coefficient, ${A}_{s}$ is the safety factor considering the tire slip, and R is the tire rolling radius.

### Analysis of cooperative control mechanism of path tracking and yaw stability

The objective of DDAV path tracking control is to make the driving position and direction as consistent as possible with the target path, and the DDAV typically realizes the target path direction tracking by controlling the vehicle’s yaw angle. In DDAV yaw stability control, the yaw angular velocity is typically considered as a control objective, and can be obtained from the definition of the yaw angle and yaw angular velocity, as follows:

10 $$\dot{{\psi }_{R}}=\gamma$$

According to Eq. (10), PFC and YSC are mutually coupled, and YSC assists PFC by controlling the vehicle yaw rate. In the yaw stability control process, the yaw rate target value design method has an important effect on whether the PFC and YSC can achieve cooperative control. The yaw rate target value designed by unilaterally considering the yaw stability improves the vehicle yaw stability. However, the PFC control accuracy may be reduced. The role of PFC is to achieve satisfactory vehicle driving performance, and the function of YSC is to ensure the safety performance of vehicle movement. In contrast, safety is the primary objective of vehicle driving, but YSC is not required when the vehicle runs stably and safely. Therefore, it is necessary to distinguish the stable state of the vehicle’s lateral movement. When the vehicle lateral movement tends to be unstable, the YSC is mainly used for stability control; when the vehicle lateral movement is stable and safe, the YSC mainly assists the PFC in improving the path tracking accuracy.

**Figure 2:**
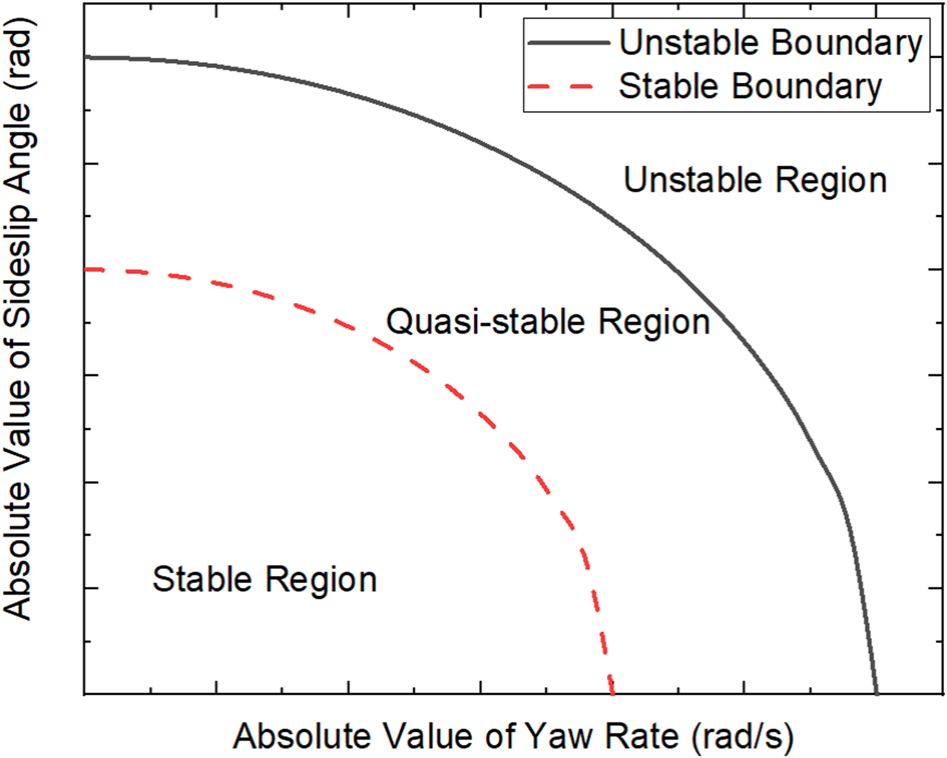
Results of dynamic boundary partitioning of lateral motion control domain.

In this study, the dynamic boundary, which introduces the pavement adhesion coefficient, is used to assess the stable state of the DDAV lateral motion and provide the vehicle stable state criterion for YSC and PFC synergistic control. The dynamic boundary is described by the yaw rate and sideslip angle to evaluate the lateral stability of the DDAV. Under general driving conditions, the lateral dynamic response of the vehicle is within the linear range and exhibits good handling stability. The steady-state response of the vehicle in the linear range is determined as the stability boundary. When the vehicle is subjected to increasingly more severe working conditions, the lateral dynamic response exhibits increasingly more obvious nonlinearity, and the vehicle is eventually exposed to lateral instability. The state wherein the tire lateral force reaches saturation and is about to lose lateral stability is determined as the unstable boundary. Both the stable boundary and unsteady boundary model introduce road adhesion coefficients such that the designed dynamic boundary can more reasonably describe the stability of the DDAV under different road conditions. As shown in Fig. 2, the stable boundary and unstable boundary constitute the dynamic boundary. The dynamic boundary divides the yaw stability control region into the stable region, quasi-stable region, and unstable region. When the vehicle is in a stable state, the vehicle exhibits good lateral stability and yaw stability control is not required. When the vehicle is in a quasi-stable state, the yaw stability of the vehicle tends to deteriorate. Therefore, yaw stability control should be carried out to prevent the vehicle from entering an unsteady state.

To introduce the road adhesion coefficient into the stable boundary of the dynamic boundary, a 2-DOF vehicle dynamics model considering the road adhesion coefficient was derived. In this paper, the new 2-DOF vehicle dynamics model is referred to as the μ-2-DOF vehicle dynamics model. Based on Dugoff tire dynamics model, the μ-2-DOF vehicle dynamics model was derived by referring to the method of establishing a linear 2-DOF vehicle dynamics model^36^. The μ-2-DOF vehicle dynamics model was used to derive the stability boundary.

According to the assumed conditions^36^, the longitudinal velocity of the vehicle is constant and the driving force of the wheel is small. Therefore, the wheel slip rate is small and the slip rate is ignored. According to Eqs. (6)–(9), the Dugoff tire dynamics model can be simplified as follows.

Tire longitudinal force:

11 $${F}_{\mathcal{X}i}=0$$

Tire lateral force:

12 $${F}_{\mathcal{Y}i}={C}_{\mathcal{Y}i}\,{\tan}\,{\alpha }_{i}f\left({\upsigma }_{i}\right)$$

Expression of $$f\left({\upsigma }_{ij}\right)$$:

13 $${\text{f}}\left({\upsigma }_{i}\right)=\left\{\begin{array}{ll}\left(2-{\upsigma }_{i}\right){\upsigma }_{i}, & \quad {\upsigma }_{i}<1\\ 1, & \quad {\upsigma }_{i}\ge 1\end{array}\right.$$

14 $${\upsigma }_{i}=\frac{\mu {F}_{zi}\left(1-{ A}_{s}R{\omega }_{i}{\tan}{\alpha }_{i}\right)}{2{\tan}{\alpha }_{i}}$$

When the vehicle dynamics response is in the linear range, the tire sideslip angle is small; therefore, the following relationship holds:

15 $${\tan}{\alpha }_{i}\approx {\alpha }_{i}$$

Equation (12) can be simplified as follows:

16 $${F}_{\mathcal{Y}i}={C}_{\mathcal{Y}i}{\alpha }_{i}f\left({\upsigma }_{i}\right)$$

Equation (14) can be simplified as follows:

17 $${\upsigma }_{i}=\frac{\mu {F}_{zi}\left(1-{ A}_{s}R{\omega }_{i}{\alpha }_{i}\right)}{2{\alpha }_{i}}$$

The 2-DOF vehicle dynamics model can be established as follows:

18 $$\sum {F}_{\mathcal{Y}}={F}_{\mathcal{Y}f}{\cos}\delta +{F}_{xf}{\sin}\delta +{F}_{\mathcal{Y}r}$$

19 $$\sum {M}_{z}=a\left({F}_{\mathcal{Y}f}{\cos}\delta +{F}_{xf}{\sin}\delta \right)+b{F}_{\mathcal{Y}r}$$

where ${F}_{\mathcal{Y}}$ is the resultant lateral force of the ground on the vehicle; ${F}_{\mathcal{Y}f}$ and ${F}_{\mathcal{Y}r}$ are the lateral force of the ground on the front and rear wheels, respectively; ${F}_{xf}$ is the longitudinal force of the front wheel. According to the above-mentioned assumptions, the longitudinal driving force and $\delta$ are small; therefore, the following relationships hold:

20 $$\left\{\begin{array}{l}{F}_{xf}\approx 0\\ {\sin}\delta \approx \delta \end{array}\right.$$

Hence, the following relationship holds:

21 $${F}_{xf}{\sin}\delta \approx 0$$

Equations (18) and (19) can be rewritten as follows:

22 $$\sum {F}_{\mathcal{Y}}={F}_{\mathcal{Y}f}{\cos}\delta +{F}_{\mathcal{Y}r}$$

23 $$\sum {M}_{z}=a{F}_{\mathcal{Y}f}{\cos}\delta +b{F}_{\mathcal{Y}r}$$

The sideslip angles of the front and rear tires are expressed as follows^36^:

24 $${\alpha }_{f}=\beta +\frac{a\gamma }{{V}_{x}}-\delta$$

25 $${\alpha }_{r}=\beta -\frac{b\gamma }{{V}_{x}}$$

According to the assumptions^36^, δ is small, therefore, ${\cos}\delta \approx 1$. By combining Eqs. (16) and (22)–(25), the $\mu$-2-DOF vehicle dynamics model can be obtained as follows:

26 $$\fancyscript{m}\left({\dot{V}}_{\mathcal{Y}}+{V}_{\mathcal{X}}\gamma \right)={C}_{\mathcal{Y}f}f\left({\upsigma }_{f}\right)\left(\beta +\frac{a\gamma }{{V}_{x}}-\delta \right)+ {C}_{\mathcal{Y}r}f\left({\upsigma }_{r}\right)\left(\beta -\frac{b\gamma }{{V}_{x}}\right)$$

27 $${I}_{z}\dot{\gamma }=a{C}_{\mathcal{Y}f}f\left({\upsigma }_{f}\right)\left(\beta +\frac{a\gamma }{{V}_{x}}-\delta \right)+b{C}_{\mathcal{Y}r}f\left({\upsigma }_{r}\right)\left(\beta -\frac{b\gamma }{{V}_{x}}\right)$$

By combining Eqs. (26) and (27), when the vehicle responds in a steady state, the yaw rate and slip angle considering the road adhesion coefficient are expressed as follows:

28 $${\gamma }_{s\mu }=\frac{{V}_{\mathcal{X}}/L}{1+{K}_{\mu }{V}_{\mathcal{X}}^{2}} \delta$$

29 $${\beta }_{s\mu }=\frac{b-\frac{am{V}_{\mathcal{X}}^{2}}{{C}_{\mathcal{Y}f}f\left({\upsigma }_{r}\right)L}}{L\left(1+{K}_{\mu }{V}_{\mathcal{X}}^{2}\right)} \delta$$

where $L$ is the wheelbase; ${K}_{\mu }$ is expressed as follows:

30 $${K}_{\mu }=\frac{m}{{L}^{2}}\left(\frac{a}{{C}_{\mathcal{Y}r}f\left({\upsigma }_{r}\right)}-\frac{b}{{C}_{\mathcal{Y}f}f\left({\upsigma }_{f}\right)}\right)$$

Here, ${\gamma }_{s\mu }$ and ${\beta }_{s\mu }$ are the proposed stability boundaries. The stability boundary considers the influence of the road adhesion characteristics and wheel speed on the tire cornering characteristics through function $f\left({\upsigma }_{i}\right)$.

In this study, the unstable boundary is defined when the lateral force of the tire reaches saturation and the vehicle is about to become laterally unstable under the condition of road adhesion. In this state, the vehicle dynamic response is strongly nonlinear, and the assumption of the stable boundary derived above is no longer valid. Hence, it is very difficult to establish the unstable boundary through theoretical derivation, and the following empirical formulas^37^ are introduced:

31 $${\gamma }_{max}=0.85\;\upmu \text{g}/{V}_{\mathcal{X}}$$

32 $${\beta }_{max}=\text{arctan}(0.02\,\upmu {\text{g}})$$

where $g$ is the acceleration of gravity; Eqs. (31) and (32) are empirical formulas proposed in^37^, and have been used as the maximum limit of the yaw rate and sideslip angle in many yaw stability control studies. In^37^, the road attachment condition of the side slip angle and the yaw rate when the tire lateral force reaches saturation is proposed and determined as the maximum limit, which coincides with the definition of the unstable boundary in this study. Therefore, ${\gamma }_{max}$ and ${\beta }_{max}$ are determined as unstable boundaries.

## Control system design

**Figure 3:**
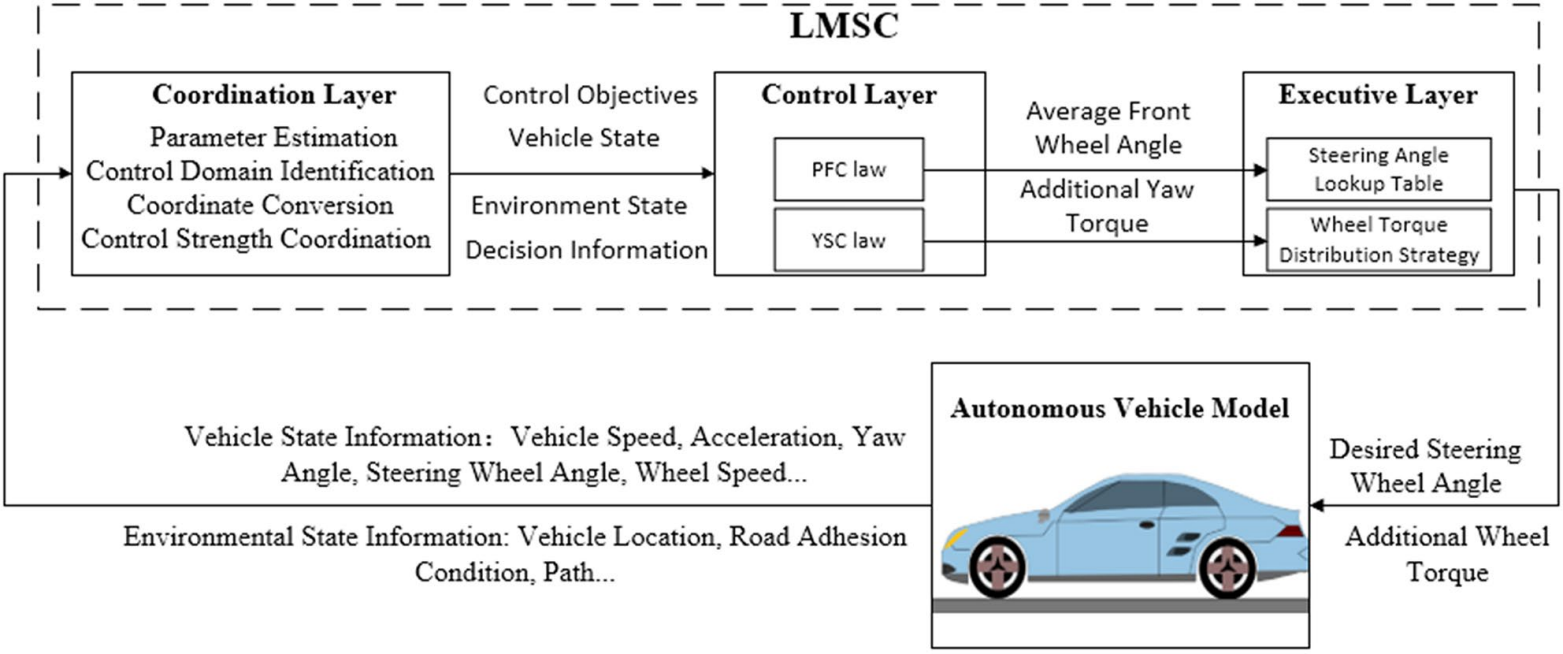
Layered control architecture of DDAV lateral motion control system.

To ensure the clarity of the control system structure and the independence of each sub-strategy function, and facilitate the system organization, LMSCS adopts the layered control architecture, which is divided into the coordination, control, and execution layers. The architecture of the designed DDAV lateral motion control system is shown in Fig. 3, where it can be seen that the coordination layer mainly completes the four tasks shown in the figure. The parameter estimation mainly completes the state parameter estimation of the tire force, center of mass slip angle, and wheel slip rate. The control domain identification layer mainly completes the dynamic boundary calculation and control domain division to form the control target. The control strength coordination mainly completes the coordination and allocation decision of the yaw stability control and path tracking control. The coordinate conversion mainly completes the conversion of GPS information into geodetic coordinate information and the further conversion into road coordinate information. The control layer mainly outputs the expected average rotation angle of the front wheels and the estimated yaw torque of the vehicle body according to the designed path tracking control law and yaw stability control law. The execution layer further calculates the steering wheel angle and additional torque of each wheel according to the calculation results of the control layer. The important part of the above-mentioned work will be described below.

### Coordination layer

The main objective of the coordination layer is to identify the control domain wherein the current vehicle state is located with a dynamic boundary, and then allocate the control strength of PFC and YSC.

#### Identification of control domain

Combined with the stable boundary and unstable boundary, the LMC control domain is identified. When the vehicle is in the stable domain, the following relationship holds:

33 $$\left|{\gamma }_{s\mu }\right|>\left|\gamma \right| \; {\text{and}} \; \left|{\beta }_{s\mu }\right|>\left|\beta \right|$$

When the vehicle is in the unstable region, the following relationship holds:

34 $$\left|\gamma \right|\ge {\gamma }_{max} \; {\text{or}} \; \left|\beta \right|\ge {\beta }_{max}$$

In other cases, the vehicles are in the quasi-stable region.

The YSC ensures that the vehicle is driven within the stability domain as much as possible. The control rules of YSC design are as follows: when the vehicle is driven within the stability domain, YSC control is not carried out. When the vehicle travels in the quasi-stable region, YSC controls the vehicle to enter the stable region; when the vehicle enters the unstable domain, it is first controlled to enter the quasi-stable domain. According to the above-mentioned rules, the objectives of YSC based on dynamic boundaries are designed as follows:

35 $${\gamma }_{d\mu }=\left\{\begin{array}{ll}{\gamma }_{s\mu }, & \quad \left|{\gamma }_{s\mu }\right|<\left|\gamma \right|<{\gamma }_{max}\\ {\gamma }_{max}\mathrm{sign}\left(\gamma \right), & \quad \left|\gamma \right|\ge {\gamma }_{max}\end{array}\right.$$

36 $${\beta }_{d\mu }=\left\{\begin{array}{ll}{\beta }_{s\mu }, & \quad \left|{\beta }_{s\mu }\right|<\left|\beta \right|<{\beta }_{max}\\ {\beta }_{max}\mathrm{sign}\left(\beta \right), & \quad\left|\beta \right|\ge {\beta }_{max}\end{array}\right.$$

#### Coordination of PFC and YSC control

Path tracking is the basic function of the DDAV for normal driving, and yaw stability is a prerequisite for safe driving. The design criterion of LMSCS is to improve the path tracking accuracy of the DDAV to ensure the vehicle yaw stability. The yaw stability of the DDAV is evaluated by the overall error of the yaw rate and sideslip angle tracking, as follows:

37 $${e}_{Y1}={\xi }_{1}\left(\gamma -{\gamma }_{d\mu }\right)+{\xi }_{2}\left(\beta -{\beta }_{d\mu }\right)$$

where ${\xi }_{1}$ and ${\xi }_{2}$ are the weight coefficients.

The yaw rate focuses on the oscillation and stability of the vehicle itself, and the sideslip angle focuses on the trajectory maintenance of the vehicle. The yaw rate also reflects the vehicle’s ability of driving in the direction of the desired path at the next moment. Controlling the yaw rate to track the ideal signal improves the vehicle yaw stability and the path tracking accuracy. To improve the comprehensive performance of DDAV lateral motion, the coordination of path tracking and yaw stability control in LMSCS is carried out as follows. The PFC is completely aimed at improving the path tracking accuracy to ensure that the vehicle is driven on the road. The YSC determines the control target according to the vehicle yaw stability state. When the yaw rate and sideslip angle are both in the stable region, the vehicle does not require yaw stability control. The YSC is assisted to improve the DDAV path tracking accuracy as the main objective, without side slip angle control. When the yaw rate is in the quasi-stable region and the slip angle is in the stable region, YSC improves the DDAV path tracking accuracy and yaw stability simultaneously, and side slip angle control is not required. In other cases, the main objective of YSC is yaw stability control. Generally, the sideslip angle is small, and even if slight disturbance exists, the sideslip angle automatically returns to a stable state. At this time, the yaw stability of the vehicle is mainly determined by the yaw rate and the yaw rate should mainly be controlled. When the sideslip angle is large, the operation of the steering wheel hardly makes the vehicle produce additional yaw moment, the vehicle is difficult to control, and the sideslip angle should mainly be controlled. With consideration to the above-mentioned factors, the values of ${\xi }_{1}$ and ${\xi }_{2}$ are designed as follows:

38 $${\xi }_{1}=1-{\xi }_{2}$$

39 $${\xi }_{2}=\left\{\begin{array}{lll}{\left(\frac{\beta -{\beta }_{s\mu }}{{\beta }_{max}-{\beta }_{s\mu }}\right)}^{2}, & \quad \left|{\beta }_{s\mu }\right|<\left|\beta \right|<{\beta }_{max} \\ 1, & \quad \left|\beta \right|\ge {\beta }_{max} \\ 0, & \quad \left|\beta \right|\le \left|{\beta }_{s\mu }\right|\end{array}\right.$$

In Eq. (39), the squaring algorithm is used such that, when $\left|\beta \right|$ is within the interval of $\left|{\beta }_{s\mu }\right|<\left|\beta \right|<{\beta }_{max}$, ${\xi }_{2}$ increases with $\left|\beta \right|$, which reflects the effect of first slowing down and then accelerating to achieve the nonlinear effect of the control distribution of the slip angle and the yaw rate. The designed coordination control law is expressed by Eq. (39). The coordination control law assesses the stable state of the DDAV lateral movement with the dynamic boundary, and then adjusts the size of ${\xi }_{1}$ and ${\xi }_{2}$ to coordinate the control force between the PFC and the YSC. Finally, PFC and YSC collaborative control is realized, and the advantages of DDAV active steering and distributed drive multi-degree-of-freedom collaborative control are activated.

### Control layer

The control layer solves the design problems of the wheel angle control law and direct yaw moment control law. The sliding mode control law is designed based on the response results of the coordination layer and GFTSM.

#### PFC law design based on GFTSM

Sliding mode design:

40 $${s}_{P1}=\dot{{e}_{P}}+{c}_{P1}{e}_{P}+{\varphi }_{1}{{e}_{P}}^{\frac{{q}_{1}}{{p}_{1}}}=0$$

where ${c}_{P1}{e}_{P}$ is the proximal attraction factor and results in the rapid exponential decay of the system state ${e}_{P}$ when it is close to the equilibrium state ${e}_{P}=0$; ${\varphi }_{1}{{e}_{P}}^{\frac{{q}_{1}}{{p}_{1}}}$ is the distal attraction factor and results in the rapid decay of the system state ${e}_{P}$ when it is far from the equilibrium state ${e}_{P}=0$; ${c}_{P1}$ and ${\varphi }_{1}$ are adjustable parameters; ${c}_{P1}>$ 0; ${\varphi }_{1}>$ 0; ${q}_{1}$ and ${p}_{1}$ are both tunable positive odd numbers; ${p}_{1}>{q}_{1}$; ${e}_{P}$ is the error expressed as follows:

41 $${e}_{P}={Y}_{R}-{Y}_{d}$$

where ${Y}_{d}$ is the expected lateral displacement of the vehicle’s center of mass in the road coordinate system; ${Y}_{d}=0$.

The exponential approaching law is adopted to enhance the system’s anti-chattering performance, as follows:

42 $$\dot{{s}_{P1}}=-{\varepsilon }_{1}\cdot sgn({s}_{P1} )-{k}_{1}{s}_{P1}$$

where ${\varepsilon }_{1}$ and ${k}_{1}$ are adjustable parameters.

When the modeling uncertainty and disturbance are large, the switching gain ${\varepsilon }_{1}$ is required to be large, which results in large chattering. To enhance the chattering resistance and avoid the excessive complication of the approaching law, the saturated function $sat\left({s}_{P1}\right)$ is used to replace the symbolic function $sgn({s}_{P1} )$^29^, and the saturated function is expressed as follows:

43 $$sat\left({s}_{P1} \right)=\left\{\begin{array}{ll}1 & \quad {s}_{P}>\Delta \\ {k}_{12}{s}_{P1} & \quad \left|{s}_{P}\right|\le \Delta \\ -1 & \quad {s}_{P}>-\Delta \end{array}\right.$$

where ${k}_{12}$ is an adjustable parameter, and ${k}_{12}=1/\Delta$.

Equation (42) can be rewritten as follows:

44 $$\dot{{s}_{P1}}=-{\varepsilon }_{1}\cdot sat({s}_{P1} )-{k}_{1}{s}_{P1}$$

According to Eq. (3), the acceleration along the ${Y}_{R}$ axis of the road coordinate system is expressed as follows:

45 $$\ddot{{Y}_{R}}={V}_{\mathcal{X}} \gamma {\sin}{\psi }_{R}+{\dot{V}}_{\mathcal{Y}}{\cos}{\psi }_{R}-{V}_{\mathcal{Y}} \gamma {\sin}{\psi }_{R}$$

According to Eq. (1), the following relationship holds:

46 $${\dot{V}}_{\mathcal{Y}}=\frac{{C}_{\mathcal{Y}f}\left(\beta +\frac{a}{V}\gamma -\delta \right)+{C}_{\mathcal{Y}r}\left(\beta -\frac{b}{V} \gamma \right)}{m}-{V}_{\mathcal{X}}\gamma$$

Combined with Eqs. (40), (41), and (44)–(46), the sliding mode control law of the average steering angle δ of the front wheel can be obtained as follows:

47 $$\delta =\frac{m\left\{{\cos}{\psi }_{R}\left[{V}_{\mathcal{X}}\gamma +\frac{\beta \left({C}_{\mathcal{Y}f}+{C}_{\mathcal{Y}r}\right)}{m}+\frac{\gamma \left({C}_{\mathcal{Y}f}a-{C}_{\mathcal{Y}r}b\right)}{m{V}_{\mathcal{X}}}\right]+{V}_{\mathcal{Y}} \gamma {\sin}{\psi }_{R}\right\}}{{C}_{\mathcal{Y}f}{\cos}{\psi }_{R}}-\frac{m\left\{{V}_{\mathcal{Y}} \gamma {\cos}{\psi }_{R}+{k}_{1}\left[{c}_{P}{e}_{P}+\dot{{e}_{P}}+\varphi {{e}_{P}}^{\frac{{q}_{1}}{{p}_{1}}}\right]\right\}+\varphi \frac{{q}_{1}}{{p}_{1}}{{e}_{P}}^{\frac{{q}_{1}-{p}_{1}}{{p}_{1}}}+{\varepsilon }_{1}\cdot sat\left({s}_{P} \right)-\ddot{{Y}_{d}}+{c}_{P}\dot{{e}_{P}}}{{C}_{\mathcal{Y}f}{\cos}{\psi }_{R}}$$

In Eq. (47), when ${e}_{P}=0$, because ${p}_{1}>{q}_{1}$ , $\frac{{q}_{1}-{p}_{1}}{{p}_{1}}<0$. Hence, ${{e}_{P}}^{\frac{{q}_{1}-{p}_{1}}{{p}_{1}}}$ cannot be calculated. In the simulation, the debugging results of parameters ${p}_{1}$ and ${q}_{1}$ are very close; therefore, it is considered that ${{e}_{P}}^{\frac{{q}_{1}-{p}_{1}}{{p}_{1}}}\approx 1$.

Global fast terminal sliding mode arrival time analysis:

According to Eq. (40), the following relationship holds:

48 $${{e}_{P}}^{\frac{{-q}_{1}}{{p}_{1}}}\frac{d{e}_{P}}{dt}+{c}_{P1}{{e}_{P}}^{1-\frac{{q}_{1}}{{p}_{1}}}=-{\varphi }_{1}$$

According to^29^, in the process of reaching the sliding mode, the time of convergence from any initial state ${e}_{Y}\left(0\right)\ne 0$ to the equilibrium state ${e}_{Y}=0$ is expressed as follows:

49 $${t}_{sP}\approx \frac{{p}_{1}}{{c}_{P1}\left({p}_{1}-{q}_{1}\right)}{\ln}\frac{{\varphi }_{1}+{c}_{P1}{{e}_{P}\left(0\right)}^{1-\frac{\mathrm{q}}{{p}_{1}}}}{{\varphi }_{1}}$$

Considering the exponential approaching law, the symbol ≈ is used in the equation. By setting parameters ${c}_{P1}$, ${\varphi }_{1}$, ${q}_{1}$, and ${p}_{1}$, the system can reach the equilibrium state in the finite time ${t}_{sP}$.

Stability analysis of PFC based on GFTSM:

The Lyapunov function is defined as follows:

50 $${V}_{P}=\frac{1}{2}{{s}_{P}}^{2}$$

Through derivation and calculation, $\dot{{V}_{P}}\le 0$; therefore, the system is stable^33^.

#### Design of YSC law based on dynamic boundary and GFTSM

Sliding mode design:

51 $${s}_{Y1}=\dot{{e}_{Y1}}+{c}_{Y1}{e}_{Y1}+{\varphi }_{2}{{e}_{Y1}}^{\frac{{q}_{2}}{{p}_{2}}}=0$$

where ${c}_{Y1}$ and ${\varphi }_{2}$ are adjustable parameters; ${c}_{Y1}>$ 0; ${\varphi }_{2}>$ 0; ${q}_{2}$ and ${p}_{2}$ are both tunable positive odd numbers; ${p}_{2}>{q}_{2}$.

By combining Eqs. (5) and (51), the additional yaw torque sliding mode control law of the vehicle can be obtained as follows:

52 $$\begin{aligned}{\Delta M}_{z} & =-a\left({F}_{\mathcal{Y}fr}+{F}_{\mathcal{Y}fl}\right){\cos}\delta -\frac{{d}_{f}}{2}\left({F}_{\mathcal{Y}fl}-{ F}_{\mathcal{Y}fr}\right){\sin}\delta +b\left({F}_{\mathcal{Y}rl}+{F}_{\mathcal{Y}rr} \right)-\frac{{d}_{f}}{2}\left({F}_{\mathcal{X}fr}-{ F}_{\mathcal{X}fl}\right){\cos}\delta \\ & \quad -a\left({F}_{\mathcal{X}fr}+ {F}_{\mathcal{X}fl}\right){\sin}\delta -\frac{{d}_{r}}{2}\left({F}_{\mathcal{X}rr}-{F}_{\mathcal{X}rl}\right)-{I}_{z}{\dot{\gamma }}_{d\mu }- \frac{{I}_{z}\left\{{\xi }_{2}\left(\dot{\beta }-{\dot{\beta }}_{d\mu }\right) +{c}_{Y1}{e}_{Y1}+{\varphi }_{2}{{e}_{Y1}}^{\frac{{q}_{2}}{{p}_{2}}} \right\}}{{\xi }_{1}}\end{aligned}$$

Global fast terminal sliding mode arrival time analysis:

According to Eq. (51), the following relationship holds:

53 $${{e}_{Y1}}^{\frac{{-q}_{2}}{{p}_{2}}}\frac{d{e}_{Y1}}{dt}+{c}_{Y1}{{e}_{Y1}}^{1-\frac{{q}_{2}}{{p}_{2}}}=-{\varphi }_{2}$$

Similarly, in the process of reaching the sliding mode, the time of convergence from any initial state ${e}_{Y1}\left(0\right)\ne 0$ to the equilibrium state ${e}_{Y1}=0$ is expressed as follows:

54 $${t}_{sY}=\frac{{p}_{2}}{{c}_{Y1}\left({p}_{2}-{q}_{2}\right)}{\ln}\frac{{\varphi }_{2}+{c}_{Y1}{{e}_{Y1}\left(0\right)}^{1-\frac{{q}_{2}}{{p}_{2}}}}{{\varphi }_{2}}$$

By setting parameters ${c}_{Y1}$, ${\varphi }_{2}$, ${q}_{2}$, and ${p}_{2}$, the system can reach the equilibrium state in the finite time ${t}_{sY}$.

Stability analysis of YSC based on GFTSM:

The Lyapunov function is defined as follows:

55 $${V}_{Y}=\frac{1}{2}{{s}_{Y1}}^{2}$$

Through derivation and calculation, $\dot{{V}_{Y}}\le 0$; therefore, the system is stable^33^.

### Executive layer

The actuator control target distribution is realized in the executive layer. The steering wheel angle is calculated according to the average front wheel angle calculated by the control layer. The additional driving torque of each wheel is calculated according to the additional yaw torque of the body calculated by the control layer.

**Figure 4:**
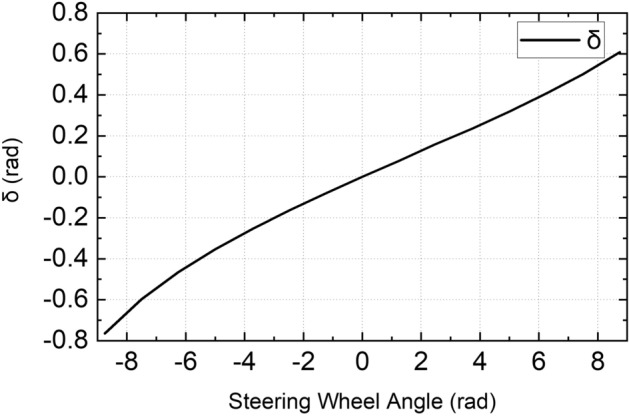
Curve of relationship between steering wheel angle and average front wheel angle.

Figure 4 shows the relationship between the DDAV steering wheel angle and the average front wheel angle. The curve was obtained by establishing the DDAV multi-body dynamics model. The PFC obtains the steering wheel angle by checking a table.

With regard to the coupling relationship of the tire forces, generally, as the wheel load increases, the maximum longitudinal or maximum lateral force becomes greater. To avoid one of the tire forces reaching saturation first, a four-wheel additional torque distribution strategy based on the wheel load is adopted. The longitudinal forces generated by the additional driving torque of the wheels are as follows:

56 $${\Delta F}_{\mathcal{x}ij}=\frac{{\Delta T}_{ij}}{R}$$

where ${\Delta T}_{ij}$ is the wheel compensation torque.

By combining Eq. (56), the compensation torques of the four wheels can be calculated as follows:

57 $${\Delta T}_{fl}=\frac{{F}_{zfl}}{{F}_{z}}\frac{{\Delta M}_{z}}{-{d}_{f}/2{\cos}\delta +a{\sin}\delta }R$$

58 $${\Delta T}_{fr}=\frac{{F}_{zfr}}{{F}_{z}}\frac{{\Delta M}_{z}}{{d}_{f}/2{\cos}\delta +a{\sin}\delta }R$$

59 $${\Delta T}_{rl}=-\frac{{F}_{zrl}}{{F}_{z}}\frac{{\Delta M}_{z}}{2{d}_{r}}R$$

60 $${\Delta T}_{rr}=\frac{{F}_{zrr}}{{F}_{z}}\frac{{\Delta M}_{z}}{2{d}_{r}}R$$

where ${F}_{z}={F}_{zfl}+{F}_{zfl}+{F}_{zfl}+{F}_{zrr}$, ${\Delta T}_{fl}$ is the left front wheel compensation torque, ${\Delta T}_{fr}$ is the right front wheel compensation torque, ${\Delta T}_{rl}$ is the left rear wheel compensation torque, ${\Delta T}_{rr}$ is the right rear wheel compensation torque, and R is the tire rolling radius.

## Demonstrative example

To verify the effectiveness and superiority of the LMSCS based on the dynamic boundary and GFTSM, a sample DDAV model was used to verify the simulation. The main parameters of the DDAV model are listed in Table 1. First, according to the data in Table 1 and Fig. 4, the vehicle dynamics model was established in LMS Imagine.Lab Amesim. Then, the control system model was established in Matlab/Simulink. The coordination layer strategy was established according to Eqs. (35)–(39), the coordination layer strategy was established according to Eqs. (35)–(39), the control layer strategy was established according to Eqs. (47) and (52), and the execution layer strategy was established according to Eqs. (57)–(60). The vehicle dynamics model established in LMS Imagine.Lab Amesim and the control system model established in Matlab/Simulink were combined to form the DDAV lateral motion control simulation model.

**Table 1:** Main DDAV parameters.

Symbol	Description	Value
m	Vehicle total mass	1430 (kg)
a	Distance from center of gravity to front axle	1.056 (m)
b	Distance from center of gravity to rear axle	1.344 (m)
${d}_{f}$	Distance between front left and right wheels	1.45 (m)
${d}_{r}$	Distance between rear left and right wheels	1.45 (m)
R	Tire rolling radius	0.29 (m)
${h}_{g}$	Height of vehicle center of gravity	0.675 (m)
${I}_{z}$	Yaw moment of inertia of vehicle	1300 (kg m^2^)
${J}_{w}$	Moment of inertia of wheel	0.85 (kg m^3^)
${C}_{f}$	Equivalent nominal front tire cornering stiffness	75,000 (N/rad)
${C}_{r}$	Equivalent nominal rear tire cornering stiffness	80,000 (N/rad)

**Figure 5:**
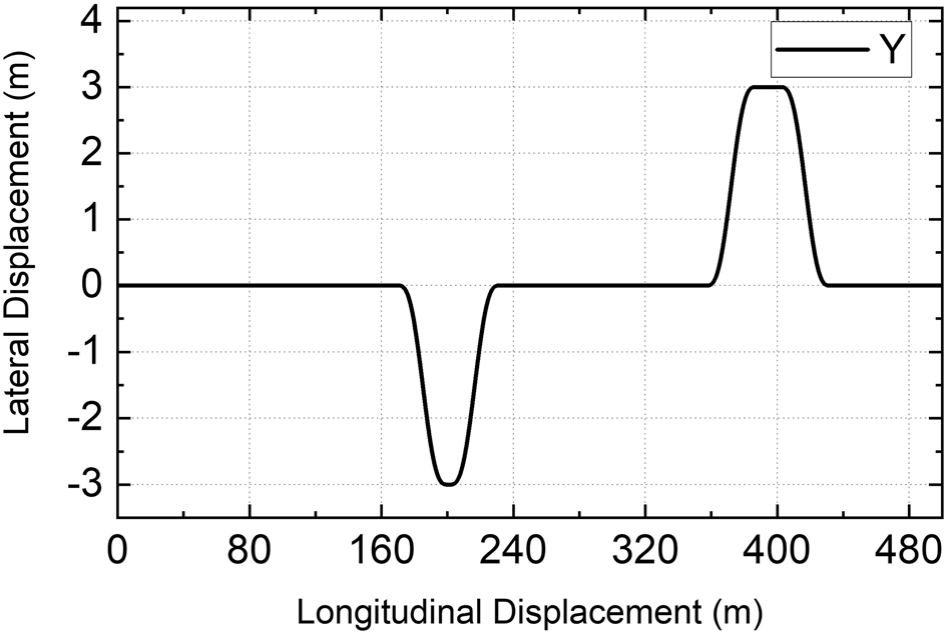
Lateral tracking displacement.

The double-side double-line shifting condition was adopted as the simulation test condition, and the length of the double-line shifting on both sides is different. The designed path is shown in Fig. 5. In the simulation process, the simulation time step is 0.01 s. To verify the robustness of LMSCS, three roads were selected for simulation analysis with two speeds for each road. The simulation conditions are listed in Table 2. The selection principle of two speeds on the same road is as follows: when LMSCS is not adopted, the DDAV will become transversely unstable at higher speed, while the DDAV will run stably at lower speed.

**Table 2:** DDAV simulation conditions.

Road type	Adhesion coefficient	Speed (km/h)
Dry road	0.8	65
		45
Wet road	0.5	55
		40
Ice road	0.15	30
		20

### Validation of LMSCS based on dynamic boundary and GFTSM

For the convenience of description, the DDAV lateral motion control system, which only adopts the PFC based on GFTSM, is considered as the Primary Lateral Motion Control System (PLMCS). By comparing the simulation results obtained for PLMCS and LMSCS, it is confirmed that LMSCS fully enables the lateral dynamic performance control potential brought by the multi-degree-of-freedom controllable advantages of the DDAV. Finally, the DDAV path tracking accuracy and yaw stability are improved simultaneously. Due to the limited space of the paper, only the data curves under some working conditions are shown below, and the data statistics under all working conditions are shown in Table 3.

**Table 3:** Summary of DDAV double line driving simulation results.

Strategy			PLMCS	LMSCS	
$\mu$	${V}_{\mathcal{X}}$(km/h)	Parameters	Range	Range	Optimization ratio (%)
0.8	65	${e}_{P}$	[− 0.021 m,0.020 m]	[− 0.022 m,0.021 m]	− 4.8
		β	[− 0.067 rad,0. 055 rad]	[− 0.030 rad,0. 030 rad]	55.2
		γ	[− 0.513 rad/s,0.577 rad/s]	[− 0.381 rad/s,0.396 rad/s]	31.4
		${V}_{\mathcal{X}}$	[17.910 m/s,18.060 m/s]	[17.849 m/s,18.060 m/s]	− 0.3
		${\delta }_{s}$	[− 2.115 m/s,2.378 m/s]	[− 2.001 rad,2.207 rad]	14.8
	45	${e}_{P}$	[− 0.030 m,0.030 m]	[− 0.029 m,0.030 m]	0.0
		β	[− 0. 009 rad, 0.009 rad]	[− 0. 006 rad, 0.006 rad]	33.3
		γ	[− 0. 255 rad/s,0.259 rad/s]	[− 0. 252 rad/s,0.254 rad/s]	1.9
		${V}_{\mathcal{X}}$	[12.470 m/s,12.500 m/s]	[12.485 m/s,12.510 m/s]	0.1
		${\delta }_{s}$	[− 1.040 m/s,1.049 m/s]	[− 0.912 m/s,0.914 m/s]	12.9
0.5	55	${e}_{P}$	[− 0.012 m,0.011 m]	[− 0.014 m,0.013 m]	− 16.7
		β	[− 0. 030 rad, 0.029 rad]	[− 0.011 rad, 0.009 rad]	63.3
		γ	[− 0. 363 rad/s,0.366 rad/s]	[− 0. 281 rad/s,0.281 rad/s]	23.2
		${V}_{\mathcal{X}}$	[15.210 m/s,15.280 m/s]	[15.131 m/s,15.280 m/s]	− 0.5
		${\delta }_{s}$	[− 1.769 m/s,1.792 m/s]	[− 1.938 m/s,1.938 m/s]	− 8.1
	40	${e}_{P}$	[− 0.024 m,0.024 m]	[− 0.023 m,0.023 m]	4.2
		β	[− 0.012 rad,0. 012 rad]	[− 0.010 rad,0.010 rad]	16.7
		γ	[− 0.227 rad/s,0.230 rad/s]	[− 0.222 rad/s,0.224 rad/s]	2.6
		${V}_{\mathcal{X}}$	[11.090 m/s,11.120 m/s]	[11.098 m/s,11.120 m/s]	0.1
		${\delta }_{s}$	[− 0.985 m/s,0.994 m/s]	[− 0.222 m/s,0.889 m/s]	10.6
0.15	30	${e}_{P}$	[− 0.005 m,0.006 m]	[− 0.008 m,0.007 m]	− 33.3
		β	[− 0.013 rad,0. 015 rad]	[− 0.014 rad,0. 015 rad]	0.0
		γ	[− 0.178 rad/s,0.179 rad/s]	[− 0.157 rad/s,0.155 rad/s]	12.3
		${V}_{\mathcal{X}}$	[8.325 m/s,8.336 m/s]	[8.312 m/s,8.336 m/s]	− 0.2
		${\delta }_{s}$	[− 0.963 m/s,0.949 m/s]	[− 1.042 m/s,1.067 m/s]	− 10.8
	25	${e}_{P}$	[− 0.007 m,0.006 m]	[− 0.007 m,0.006 m]	0.0
		β	[− 0.024 rad,0. 025 rad]	[− 0.024 rad,0. 025 rad]	0.0
		γ	[− 0.141 rad/s,0.141 rad/s]	[− 0.139 rad/s,0.139 rad/s]	1.4
		${V}_{\mathcal{X}}$	[6.938 m/s,6.947 m/s]	[6.939 m/s,6.947 m/s]	0.0
		${\delta }_{s}$	[− 0.796 m/s,0.799 m/s]	[− 0.784 m/s,0.788 m/s]	1.4

**Figure 6:**
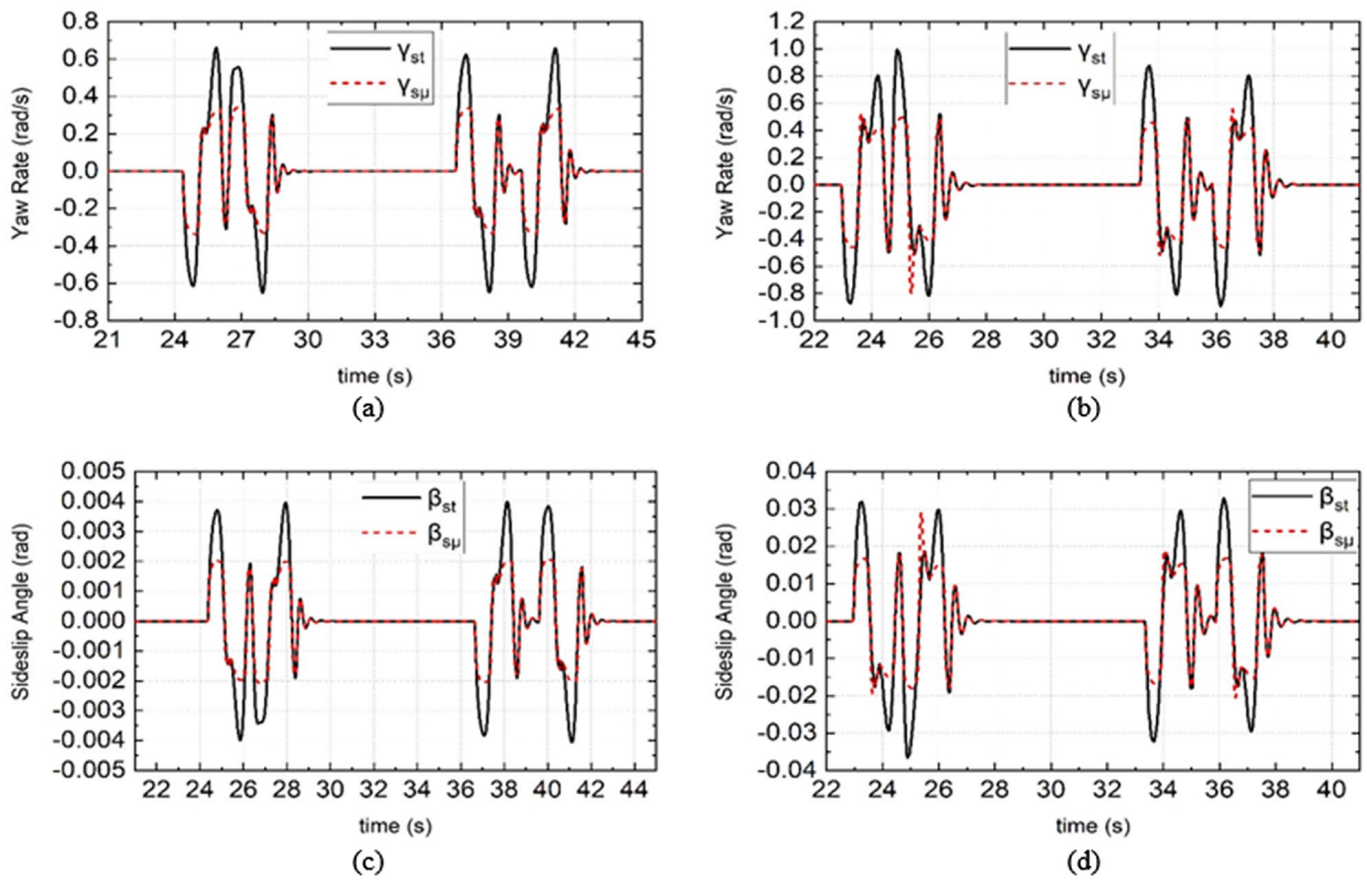
Simulation results of stability boundary under different road adhesion coefficient and different vehicle speed. (**a**) and (**c**) are the simulation results of DDAV running at 55 km/h on wet road, (**b**) and (**d**) are the simulation results of DDAV running at 65 km/h on dry road.

When PLMCS is used, the estimated DDAV ${\beta }_{st}$, ${\gamma }_{st}$, ${\beta }_{s\mu }$, and ${\gamma }_{s\mu }$ are as shown in Fig. 6; ${\beta }_{st}$ and ${\gamma }_{st}$ were calculated by the traditional 2-DOF vehicle dynamics model. By observing the peak of each parameter in Fig. 6, it is seen that, under different road adhesion coefficients, when the DDAV runs under the instability condition, ${\beta }_{st}> {\beta }_{s\mu }$ and ${\gamma }_{st}>{\gamma }_{s\mu }$. When the DDAV runs under unfavorable conditions, the vehicle dynamic response exhibits strong nonlinearity, and ${\beta }_{s\mu }$ and ${\gamma }_{s\mu }$ are smaller than ${\beta }_{st}$ and ${\gamma }_{st}$, which indicates that the designed dynamic boundary considers the influence of the road adhesion characteristics and wheel speed on the vehicle’s yaw stability, and the stability region decreases. The effectiveness of the stability boundary design considering the road adhesion coefficient was verified. The introduction of the dynamic boundary of the road adhesion coefficient helps to more accurately recognize the steady state of the vehicle’s lateral movement.

**Figure 7:**
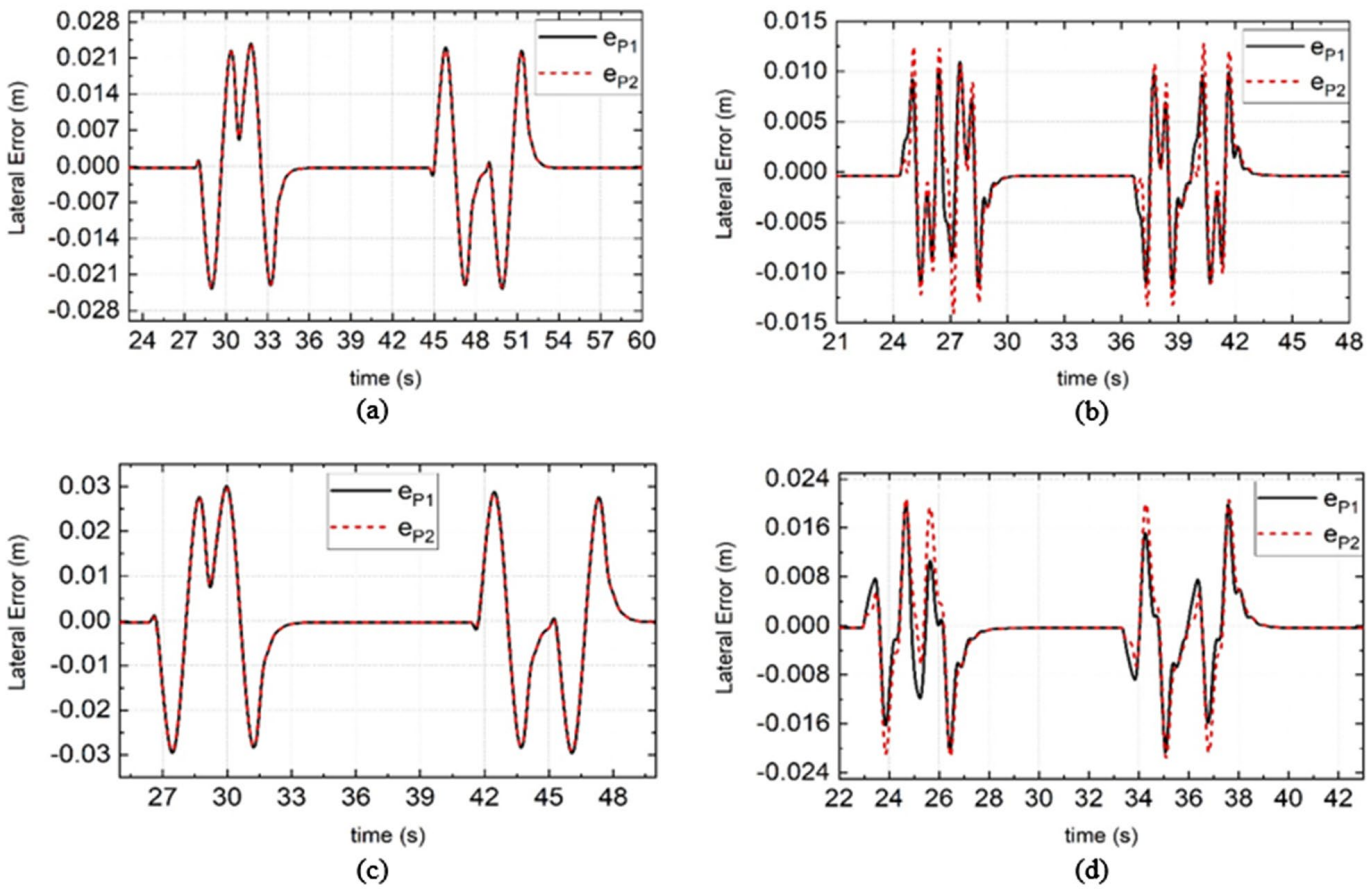
Simulation results of lateral tracking errors under different road adhesion coefficient and different vehicle speed. (**a**) Is the simulation results of DDAV running at 40 km/h on wet road, (**b**) is the simulation results of DDAV running at 55 km/h on wet road, (**c**) is the simulation results of DDAV running at 45 km/h on dry road, (**d**) is the simulation results of DDAV running at 65 km/h on dry road.

**Figure 8:**
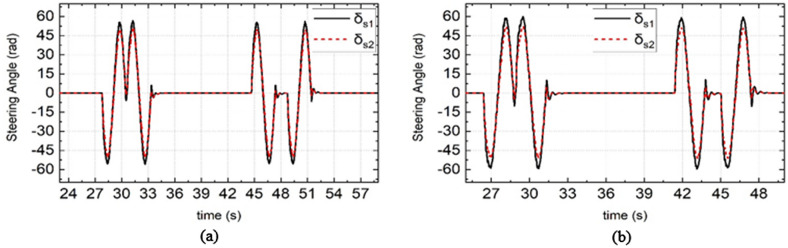
Simulation results of steering wheel angle under different road adhesion coefficient and different vehicle speed. (**a**) Is the simulation results of DDAV running at 40 km/h on wet road, (**b**) is the simulation results of DDAV running at 45 km/h on dry road.

**Figure 9:**
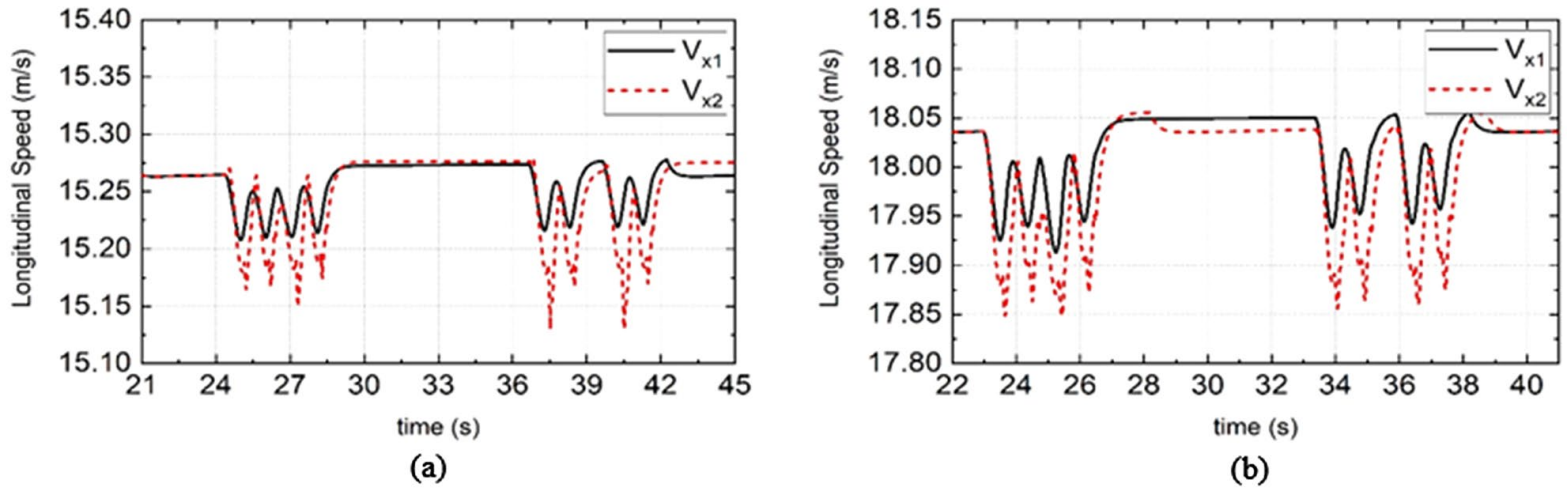
Simulation results of longitudinal speed under different road adhesion coefficient and different vehicle speed. (**a**) Is the simulation results of DDAV running at 55 km/h on wet road, (**b**) is the simulation results of DDAV running at 65 km/h on wet road.

As shown in Figs. 7, 8, and 9, when PLMCS is used, ${e}_{P1}$ is the lateral position error of DDAV path tracking, ${\delta }_{s1}$ is the steering wheel angle, and ${V}_{\mathcal{X}1}$ is the longitudinal speed. When LMSCS is used, the DDAV response results are presented as ${e}_{P2}$, ${\delta }_{s2}$, and ${V}_{\mathcal{X}2}$ in Figs. 7, 8, and 9. According to Fig. 7a,c and Fig. 8, when DDAV runs stably, YSC reduces the path tracking error or steering wheel angle, which improves the path tracking performance of DDAVs. According to Fig. 9, when PLMCS or LMSCS is applied, the longitudinal speed fluctuates within a small range centered on the target speed. Compared with PLMCS, LMSCS does not affect the time required for the control system to reach stability. The proposed control system based on the dynamic boundary and GFTSM has little influence on the longitudinal motion of DDAVs.

**Figure 10:**
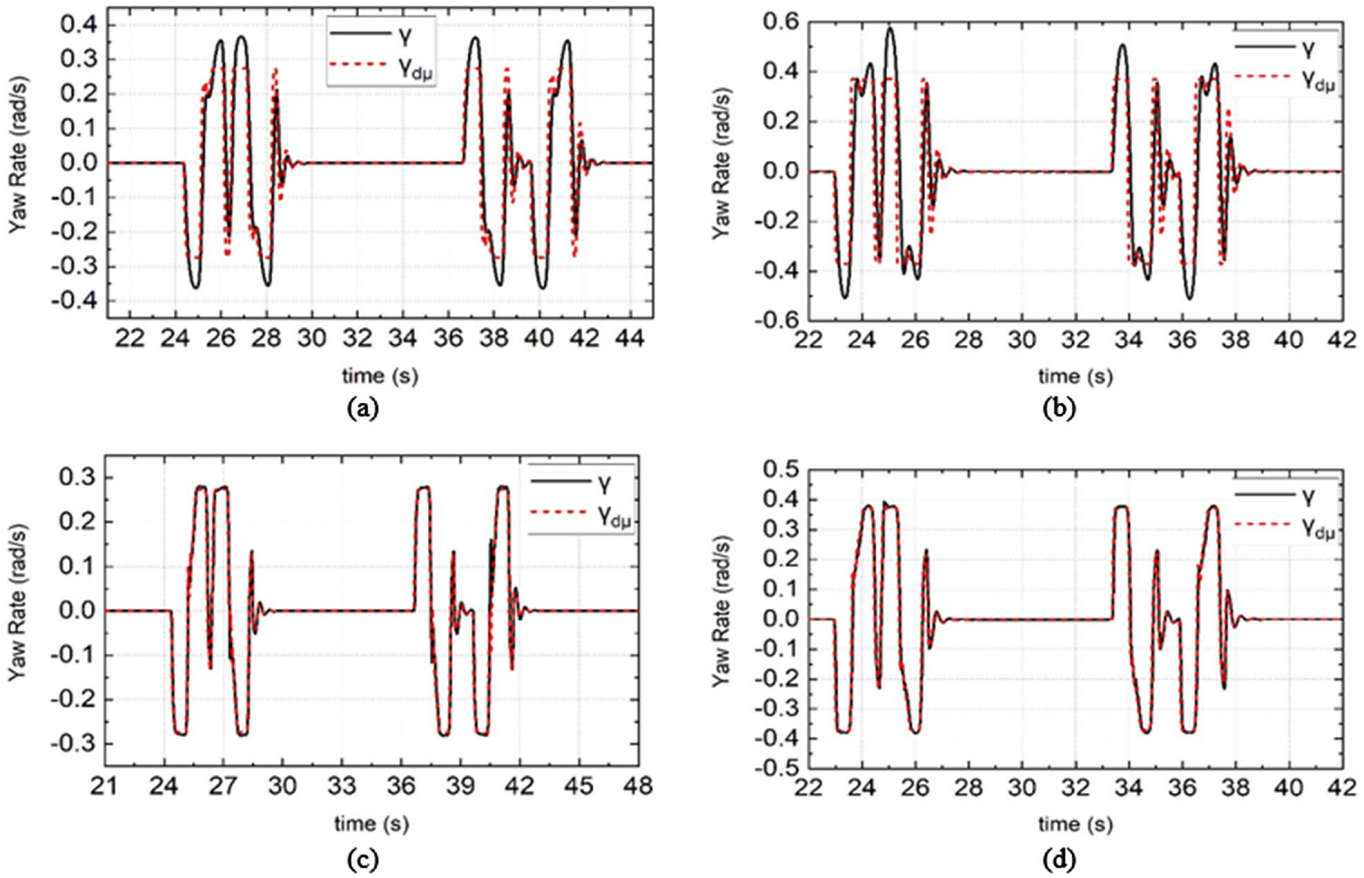
Simulation results of yaw rate under different road adhesion coefficient and different vehicle speed. (**a**) and (**c**) are the simulation results of DDAV running at 55 km/h on wet road, (**b**) and (**d**) are the simulation results of DDAV running at 65 km/h on dry road.

**Figure 11:**
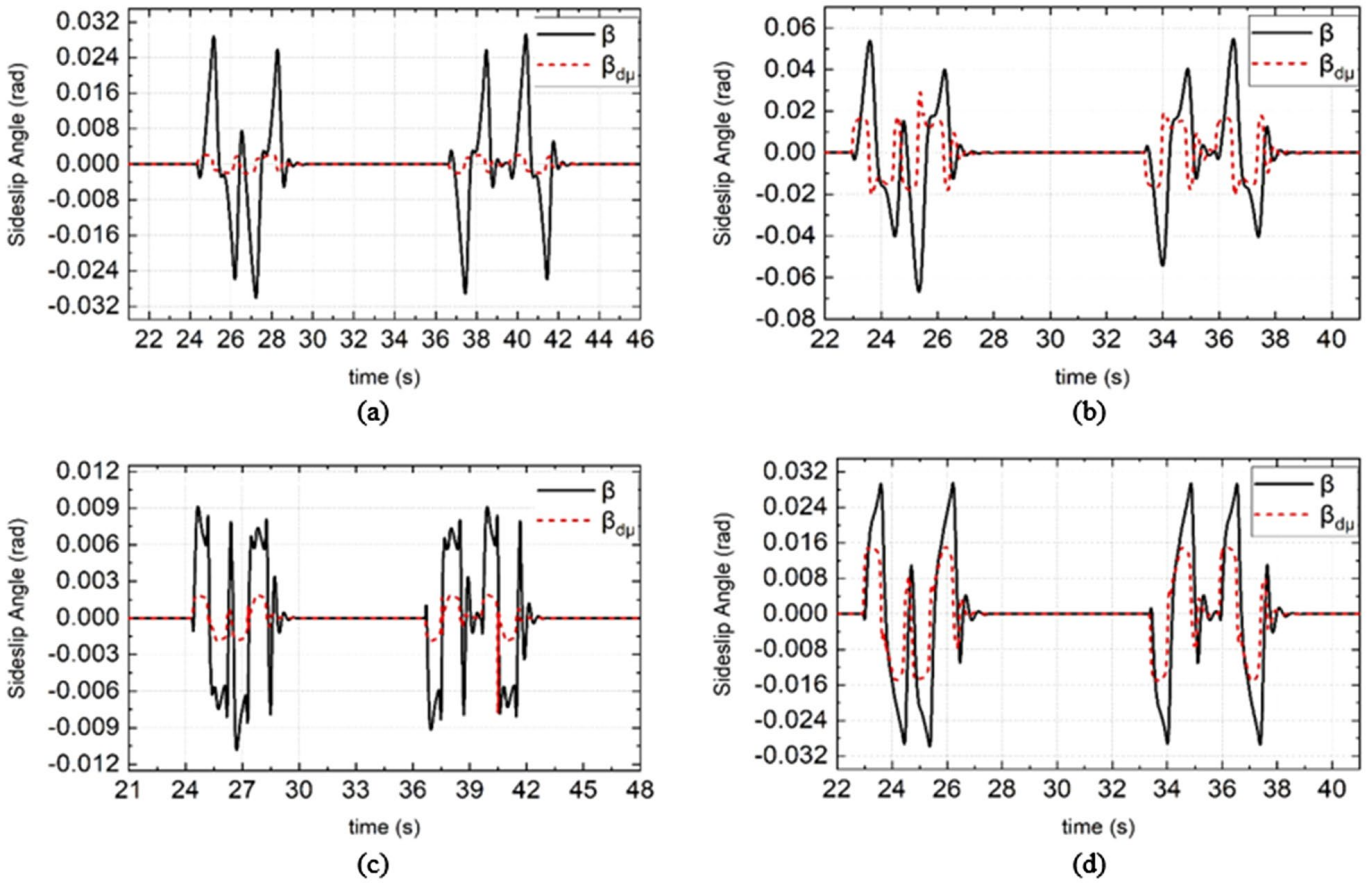
Simulation results of sideslip angle under different road adhesion coefficient and different vehicle speed. (**a**) and (**c**) are the simulation results of DDAV running at 55 km/h on wet road, (**b**) and (**d**) are the simulation results of DDAV running at 65 km/h on dry road.

When DDAV is driving in unstable conditions, when PLMCS is used, the yaw rate γ and sideslip angle β are shown in Figs. 10a,b and 11a,b, and when LMSCS is used, the results are shown in Figs. 10c,d and 11c,d. When LMSCS is adopted, the sideslip angle is greatly reduced, and the yaw rate almost never exceeds the target yaw rate ${\gamma }_{d\mu }$.Therefore, the yaw stability of the DDAV is improved and the lateral stability of the DDAV is avoided in the unstable region, which demonstrates the effectiveness of YSC.

.

By sorting and comparing the simulation results obtained using PLMCS and LMSCS, respectively, the summary of the simulation is presented in Table 3. The optimized ratios in the table are calculated relative to the PLMCS control results, and according to the larger absolute value of the upper and lower boundaries of the corresponding parameter change range. For example, ${e}_{P}$ obtained under the 65 km/h condition is presented in the table. Because abs(− 0.020) < abs(0.021) and abs(− 0.021) < abs(− 0.022), the optimization ratio is [abs(0.021) − abs(0.022)]/abs(0.021) $\approx$ − 4.8%. By analyzing the data in Table 2, the following conclusions can be drawn: (1) Under different road adhesion conditions, LMSCS significantly reduces the yaw velocity, sideslip angle, and lateral acceleration when the DDAV is about to become transversely unstable. The maximum optimization ratio of the sideslip angle of the centroid reaches 63.3%. The optimal proportion of the yaw velocity is 31.4%. Therefore, under different road adhesion conditions, when the DDAV is about to lose lateral stability, LMSCS effectively improves the yaw stability and lateral stability. (2) Under different road adhesion conditions, LMSCS reduces the lateral error of path tracking and the steering wheel angle when the DDAV maintains stable driving speed. Therefore, under different road adhesion conditions, LMSCS improves the path tracking performance when the DDAV runs stably. (3) Under different road adhesion conditions and at different vehicle speeds, LMSCS improves the lateral stability and path tracking performance of the DDAV, and its influence on the longitudinal vehicle tracking performance is less than 1%.

The above conclusions reveal that the DDAV lateral motion cooperative control method based on the proposed dynamic boundary fully activates the advantages of DDAV active steering and distributed drive multi-degree-of-freedom cooperative control. Thus, LMSCS improves the path tracking performance and vehicle yaw stability simultaneously.

### LMSCS superiority verification based on dynamic boundary and GFTSM

To date, many studies have successfully applied the lateral motion control method based on the Quasi Sliding Mode (QSM) for the lateral motion control of autonomous vehicles or distributed driving vehicles, and the effectiveness of the algorithm has been verified by simulation or tests on actual vehicles. In this study, the vehicle lateral motion control system based on QSM was considered as the Lateral Motion Traditional Control System (LMTCS). To further verify the superiority of LMSCS, the simulation results of LMSCS and LMTCS were compared and analyzed.

Through a calculation process similar to that of GFTSM-based PFC, the front wheel average angle control law in LMTCS can be obtained as follows:

61 $$\begin{aligned} {\delta }_{c} & =\frac{m\left\{\ddot{{Y}_{d}}+\frac{{\cos}{\psi }_{R}}{m}\left[{V}_{\mathcal{X}}\gamma +\frac{\beta \left({C}_{f}+{C}_{r}\right)+\gamma \left({C}_{f}a+{C}_{r}b\right)}{{V}_{\mathcal{X}}}\right]+{V}_{\mathcal{Y}} \gamma {\sin}{\psi }_{R}\right\}}{{C}_{f}{\cos}{\psi }_{R}} \\ & \quad -\frac{m\left[{V}_{\mathcal{X}}\gamma {\cos}{\psi }_{R}+{c}_{P2}\left({e}_{P} +\dot{{e}_{P}}\right)+{k}_{2}\left(\dot{Y}-\dot{{Y}_{d}}\right)+{\varepsilon }_{2}\cdot sat({s}_{P2} )\right]}{{C}_{f}{\cos}{\psi }_{R}} \end{aligned}$$

where the sliding mode ${s}_{P2}=\dot{{e}_{P}}+{c}_{P2}{e}_{P}$; ${c}_{P2}$, ${k}_{2}$, and ${\varepsilon }_{2}$ are adjustable parameters.

The YSC in LMTCS also adopts the exponential approach law, and the saturated function $sat\left({s}_{P1}\right)$ replaces the symbolic function $sgn({s}_{P1})$ in the same manner as Eq. (32). Through a calculation process similar to that of YSC based on GFTSM, the vehicle’s additional yaw torque sliding mode control law can be obtained as follows:

62 $$\begin{aligned}{\Delta M}_{zc} & =\frac{-\left\{{\varepsilon }_{3}\cdot sat\left({s}_{Y2} \right)+{k}_{3}\left[{\xi }_{4}\left(\beta -{\beta }_{d}\right)+{c}_{Y2}{e}_{Y2}-{\xi }_{3}\left(\dot{\gamma }+\mathrm{A}\right)\right]\right\}}{\left({c}_{Y2+}{k}_{3}\right)\frac{{\xi }_{3}}{{I}_{z}}} \\ & \quad - \frac{\left\{{\xi }_{4}\ddot{{e}_{Y2}}+{c}_{Y2}\left[{\xi }_{4}\left(\beta -{\beta }_{d}\right)-{\xi }_{3}\left(\dot{\gamma }+\mathrm{A}\right)\right]\right\}}{\left({c}_{Y2+}{k}_{3}\right)\frac{{\xi }_{3}}{{I}_{z}}}\end{aligned}$$

where the sliding mode ${s}_{Y2}=\dot{{e}_{Y2}}+{c}_{Y2}{e}_{Y2}$; ${e}_{Y2}={\xi }_{3}\left(\gamma -{\gamma }_{dt}\right)+{\xi }_{4}\left(\beta -{\beta }_{dt}\right)$; ${c}_{Y2}$, ${k}_{3}$, and ${\varepsilon }_{3}$ are adjustable parameters; ${\xi }_{3}$ and ${\xi }_{4}$ are weight coefficients and are determined by the same method as ${\xi }_{1}$ and ${\xi }_{2}$; ${\gamma }_{dt}$ and ${\beta }_{dt}$ are expressed as follows:

63 $${\gamma }_{dt}=\left\{\begin{array}{ll}{\gamma }_{st}, & \quad \left|{\gamma }_{st}\right|<\left|\gamma \right|<{\gamma }_{max}\\ {\gamma }_{max}\mathrm{sign}\left(\delta \right), & \quad \left|\gamma \right|\ge {\gamma }_{max}\end{array}\right.$$

64 $${\beta }_{dt}=\left\{\begin{array}{ll}{\beta }_{st}, & \quad \left|{\beta }_{st}\right|<\left|\beta \right|<{\beta }_{max}\\ {\beta }_{max}\mathrm{sign}\left(\delta \right), & \quad \left|\beta \right|\ge {\beta }_{max}\end{array}\right.$$

Parameter A is expressed as follows:

65 $$\begin{aligned} A & =\frac{\frac{{d}_{r}}{2}\left({F}_{\mathcal{X}rl}-{F}_{\mathcal{X}rr}\right)+\frac{{d}_{f}}{2}\left[\left({F}_{\mathcal{X}fl}-{F}_{\mathcal{X}fr}\right)\mathit{cos}\delta -\left({F}_{\mathcal{Y}fl}-{F}_{\mathcal{Y}fr}\right)\mathit{sin}\delta \right]}{{I}_{z}} \\ & \quad -\frac{a\left[\left({F}_{\mathcal{Y}fr}+{F}_{\mathcal{Y}fl}\right)\mathit{cos}\delta +\left({F}_{\mathcal{X}fr}+{F}_{\mathcal{X}fl}\right)\mathit{sin}\delta \right]+ b\left({F}_{\mathcal{Y}rl}+{F}_{\mathcal{Y}rr} \right)}{{I}_{z}} \end{aligned}$$

The DDAV lateral dynamics control performance can be evaluated by the control errors ${e}_{P}$, ${e}_{\gamma }=\gamma -{\gamma }_{d}$, ${e}_{\beta }=\beta -{\beta }_{d}$, and control actuation $\Delta X$, where ${\gamma }_{d}$ represents ${\gamma }_{dt}$ or ${\gamma }_{du}$, ${\beta }_{d}$ represents ${\beta }_{dt}$ or ${\beta }_{du}$, and $\Delta X$ represents $\delta$ or ${\Delta M}_{z}$. To compare the dynamic performance of a DDAV during double-line shifting, the control error and control actuation torque are processed as follows^27^.

Integrate the absolute value of the error in the simulation time period:

66 $$IAE={\int }_{t1}^{t2}\left|e(t)\right|dt$$

The absolute value of the error is weighted by time and integrated within the simulation time period:

67 $$ITAE={\int }_{t1}^{t2}t\left|e(t)\right|dt$$

Integrate the absolute value of control actuation within the simulation period:

68 $$IACA={\int }_{t1}^{t2}\left|\Delta X\right|dt$$

In Eqs. (66) and (67), $e(t)$ denotes ${e}_{P}$, ${e}_{\gamma }$, or ${e}_{\beta }$; $t1$ = 42 s; $t2$ = 55 s; when $e\left(t\right)={e}_{P}$, ${IAE}_{p}$ and ${ITAE}_{p}$ exist; when $e\left(t\right)={e}_{\gamma }$, ${IAE}_{\gamma }$ and ${ITAE}_{\gamma }$ exist; when $e\left(t\right)={e}_{\beta }$, ${IAE}_{\beta }$ and ${ITAE}_{\beta }$ exist. In Eq. (68), $\Delta X$ denotes $\delta$ or ${\Delta M}_{z}$; when $\Delta X=\delta$, ${IACA}_{\delta }$ exists; when $\Delta X={\Delta M}_{z}$, ${IACA}_{M}$ exists.

To fully describe the control performance of LMTCS and LMSCS and carry out a reasonable evaluation, ITAE is applied to supplement the evaluation effect of IAE, because, although IAE reflects the sum of control errors in the control process, it cannot reflect the speed of control convergence^38^.

The simulation results reveal that both LMTCS and LMSCS realize DDAV lateral motion control. The results of various evaluation indices obtained by simulation are presented in Table 4. By analyzing the data in Table 4 and comparing the evaluation indices of ${e}_{P}$ and $\delta$, it can be seen that the ability to reduce the path tracking error is LMSCS < LMTCS, and the ability to demand the steering wheel angle is LMTCS > LMSCS; therefore, the path tracking ability is LMSCS $\approx$ LMTCS. By comparing ${e}_{\beta }$, ${e}_{\gamma }$, and ${\Delta M}_{z}$, it can be seen that the yaw speed and side angle tracking ability is LMSCS > LMTCS, and the additional torque demand ability is LMTCS > LMSCS; therefore, the yaw stability control ability is LMSCS > LMTCS. Generally, LMTCS and LMSCS have similar path tracking control ability, and LMSCS is superior to LMTCS in terms of yaw stability control. Therefore, the LMSCS based on the dynamic boundary and GFTSM has better comprehensive DDAV lateral motion control compared with LMTCS based on QSM.

**Table 4:** Evaluation index statistics of lateral movement control.

Strategy			LMSCS			LMTCS		
$\mu$	${V}_{\mathcal{X}}$(km/h)	Parameters	IAE	ITAE	IACA	IAE	ITAE	IACA
0.8	65	${e}_{P}$	0.081	2.487	–	0.08	2.454	–
		${e}_{\gamma }$	0.226	6.877	–	0.23	6.999	–
		${e}_{\beta }$	0.078	2.356	–	0.078	2.359	–
		$\delta$	–	–	0.487	–	–	0.493
		${\Delta M}_{z}$	–	–	20,611	–	–	20,800
	45	${e}_{P}$	0.197	7.305	–	0.189	7.017	–
		${e}_{\gamma }$	0.1817	6.658	–	0.195	7.14	–
		${e}_{\beta }$	0.038	1.386	–	0.037	1.363	–
		$\delta$	–	–	0.3635	–	–	0.3661
		${\Delta M}_{z}$	–	–	15,516	–	–	18,478
0.5	55	${e}_{P}$	0.061	2.038	–	0.06	2.006	–
		${e}_{\gamma }$	0.179	6.053	–	0.204	6.865	–
		${e}_{\beta }$	0.045	1.5	–	0.047	1.557	–
		$\delta$	–	–	0.524	–	–	0.522
		${\Delta M}_{z}$	–	–	21,334	–	–	21,873
	40	${e}_{P}$	0.178	7.085	–	0.168	6.688	–
		${e}_{\gamma }$	0.138	5.429	–	0.146	5.751	–
		${e}_{\beta }$	0.038	1.518	–	0.038	1.506	–
		$\delta$	–	–	0.394	–	–	0.396
		${\Delta M}_{z}$	–	–	14,380	–	–	16,775
0.15	30	${e}_{P}$	0.056	2.767	–	0.049	2.369	–
		${e}_{\gamma }$	0.056	2.627	–	0.061	2.835	–
		${e}_{\beta }$	0.026	1.226	–	0.026	1.234	–
		$\delta$	–	–	0.5244	–	–	0.522
		${\Delta M}_{z}$	–	–	10,099	–	–	11,832
	25	${e}_{P}$	0.061	3.042	–	0.051	2.457	–
		${e}_{\gamma }$	0.043	2.2	–	0.047	2.349	–
		${e}_{\beta }$	0.03	1.516	–	0.03	1.514	–
		$\delta$	–	–	0.566	–	–	0.567
		${\Delta M}_{z}$	–	–	8104	–	–	9516

## Conclusion

This paper proposes a DDAV lateral motion cooperative control method based on the dynamic boundary and GFTSM. The dynamic boundary of the proposed method considers the influence of road adhesion and wheel speed on the tire sidetracking characteristics, which is beneficial for accurately identifying the stable state of vehicle lateral motion under different road conditions and dividing the control domain. The PFC and YSC cooperative mechanism was designed based on the recognition results obtained for the vehicle’s lateral motion stability. The PFC and YSC control laws based on GFTSM were designed. The simulation results confirm that the designed control method improves the vehicle path tracking when the DDAV runs stably, and improves the vehicle’s yaw stability when the DDAV runs under unfavorable conditions and is about to become unstable. Compared with the DDAV lateral motion control system based on the traditional sliding mode algorithm, the DDAV lateral motion control system has better comprehensive DDAV lateral motion control performance. In future work, the dynamic boundary will be extended, and additional DDAV motion stability state evaluation parameters will be introduced based on the yaw velocity and sideslip angle such that the dynamic boundary can more comprehensively and accurately identify the vehicle stability state and provide state criteria for vehicle motion control.
